# Potential for low-forage diets to maintain milk production in forage-limited situations

**DOI:** 10.3168/jdsc.2023-0407

**Published:** 2023-11-17

**Authors:** Ariana Negreiro, Adam L. Lock

**Affiliations:** Department of Animal Science, Michigan State University, East Lansing 48824

## Abstract

We determined the effect of feeding diets similar in neutral detergent fiber (NDF), starch, and crude protein (CP) with different amounts of forage on the yields of milk and milk components of mid-lactation dairy cows. Thirty-two Holstein cows (132 ± 68 d in milk) were used in a crossover design with 2 consecutive 28-d periods, with sample and data collection during the final 5 d of each period. Treatment diets were (1) control diet (CON) containing high forage (55.5% diet dry matter [DM]; forage NDF 19.2% diet DM) and no supplemental fatty acids or AA; and (2) low-forage (LF) diet containing low forage (36.6% diet DM; forage NDF 12.7% diet DM), including supplemental fat (1.43% diet DM; 82% C16:0-enriched supplement) and rumen-protected methionine and lysine. Diets were balanced for similar NDF (∼30.2% diet DM), starch (∼26.7% diet DM), and CP (∼16.2% diet DM). There was no effect of treatment on milk yield, milk fat content, or body weight. Compared with CON, LF increased DM intake (30.8 vs. 31.8 kg/d), milk fat yield (1.78 vs. 1.84 kg/d), milk protein yield (1.47 vs. 1.56 kg/d), milk protein content (3.24% vs. 3.41%), energy-corrected milk (48.3 vs. 50.2 kg/d), and body condition score (3.2 vs. 3.3). Our results demonstrate that feeding a low-forage diet supplemented with a C16:0-enriched fatty acid supplement and AA increased DM intake and the yields of milk fat and protein, without changes in body weight. The effect of a low-forage diet without supplemental fatty acids and AA was not tested.

## Highlights

• The low-forage diet increased DMI, feed efficiency, and the yields of milk fat and protein.
• The low-forage diet decreased forage DMI by 5.5 kg/cow per day.

## Graphical Abstract

**Figure:**
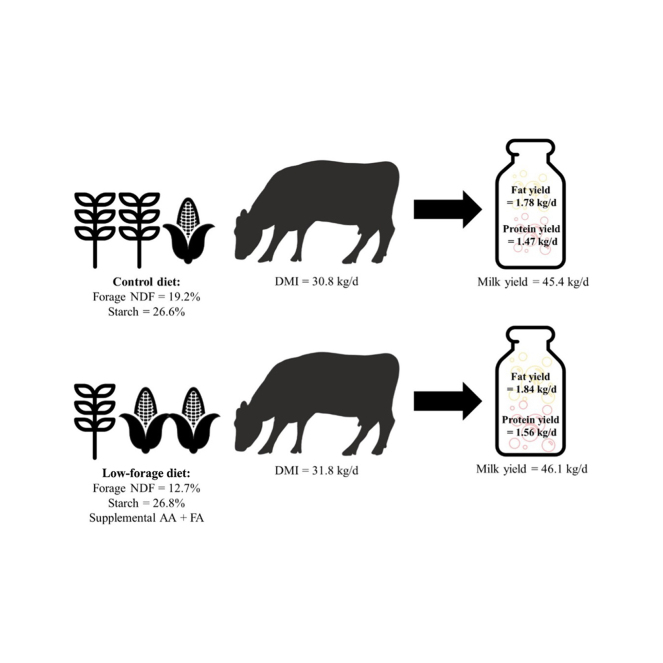
**Summary** With changes in availability of high-quality forages due to unusual cropping seasons or geographic location, it is important to explore alternative fiber sources and lower forage diets. High-producing, mid-lactation multiparous Holstein cows were fed a control diet containing typical Midwestern forage inclusion levels (19% forage neutral detergent fiber [NDF]) or a low-forage diet (13% forage NDF) plus a C16:0-enriched fatty acid supplement and bypass methionine and lysine. Cows fed the low-forage diet had increased dry matter intake (DMI) and yields of milk fat and protein compared with control. Additionally, the low-forage diet decreased the yields of de novo fatty acids and increased the yields of mixed and preformed milk fatty acids.

With changes in availability of high-quality forages due to unusual cropping seasons, geographic location, or increasing herd size with fixed forage inventories, it is important to explore the effects of low-forage diets and alternative fiber sources in dairy nutrition. Previous studies have taken many different approaches to formulating low-forage diets, such as increasing the proportion of byproducts in low-forage diets with low starch content (Hall and Chase, 2014), comparing low-starch diets with different amounts of forage (Farmer et al., 2014), altering the starch:NDF ratio in low- and high-forage diets (Pereira and Armentano, 2000), or comparing the effects of nonforage fiber sources on nutrient digestibility (Clark and Armentano, 1997; Mooney and Allen, 1997). However, the majority of studies have investigated the effects of low-forage diets in lower producing cows (∼30 to 35 kg/d milk yield; Clark and Armentano, 1997; Hall and Chase, 2014), and it is important to examine the effects of low-forage diets in high-producing cows with greater nutrient requirements and DMI.

Many studies have observed an increase in DMI with low-forage diets (Weiss and Pinos-Rodríguez, 2009; Farmer et al., 2014) associated with a higher rumen turnover rate due to the higher NDF digestibility of nonforage fiber sources (Allen, 2000). To mitigate the negative effects of excessive starch fermentation under these conditions, many studies decreased starch content (Pereira and Armentano, 2000). However, sufficient ruminal starch fermentation is required to support microbial protein yield (Allen, 2000), with high-producing cows responding more positively to an increase in dietary starch concentration than low-producing cows (Boerman et al., 2015). Milk protein yield is directly linked to energy intake (Doepel et al., 2004) and increasing the supply of AA, specifically methionine and lysine, to the mammary gland can increase milk protein yield (Schwab and Broderick, 2017). Therefore, to support milk production in low-forage diets our study used rumen-protected methionine and lysine supplementation in addition to supplemental fatty acids (**FA**).

In contrast, various studies have investigated the effects of FA supplementation in low-forage diets. Weiss and Pinos-Rodríguez (2009) fed high-producing cows (average ∼46 kg of milk per day) low- and high-forage diets with similar total NDF (∼32% of diet DM) and starch (∼29% of diet DM) content with or without a FA supplement containing 45% C18:0 and 28% C16:0. In the low-forage diet, the FA supplement (2.3% of DM) increased milk yield versus the low-forage diet without supplemented FA (Weiss and Pinos-Rodríguez, 2009). Recent research suggests that dairy cows have different metabolic and production responses when fed different combinations of C16:0, C18:0, and *cis*-9 C18:1 (de Souza et al., 2018, 2019). A large body of evidence has shown that C16:0-enriched (>80% C16:0) supplementation to diets with typical dietary NDF for the Midwest (42–45% of diet DM) consistently increases milk production and NDF digestibility compared with non-FA supplemented control diets (dos Santos Neto et al., 2021b) and diets supplemented with other supplements with different blends of FA (de Souza et al., 2018; Western et al., 2020). Therefore, the objective of our present study was to evaluate the effects of a low-forage diet balanced for total NDF, starch, and CP on production of high-producing, mid-lactation dairy cows. Our hypothesis was that a low-forage diet containing a C16:0-enriched FA supplement and rumen-protected methionine and lysine would maintain or surpass the yields of milk and milk components compared with a typical Midwestern diet and allow for reductions in forage usage depending on geographic and economic needs.

Experimental procedures were approved by the Michigan State University Institutional Animal Care and Use Committee. Thirty-two multiparous, mid-lactation Holstein cows (mean ± SD: 132 ± 68 DIM, 50.8 ± 4.4 kg/d milk, 702 ± 54 kg of BW) were housed at the Michigan State University Dairy Cattle Teaching and Research Center. The study was completed from October to December 2019. All animals received a common diet containing typical Midwestern forage levels during a 7-d preliminary period and then were randomly assigned to treatment sequences in a crossover design experiment with 2 consecutive 28-d periods.

Treatments were a (1) control (**CON**) diet containing 19.2% forage NDF and no supplemental FA or supplemental AA and (2) low-forage (**LF**) diet containing 12.7% forage NDF, including supplemental FA (1.5% diet DM; 82% C16:0-enriched supplement) and rumen-protected methionine and lysine (0.1% diet DM and 0.2% diet DM, respectively). Although the diets differed in fiber and starch sources, they were formulated to contain similar total NDF, starch, and CP. The diets differed in RUP and RDP content as a result of keeping CP constant with the addition of supplemental AA to the LF diet. The ingredient and nutrient composition of the diets fed as TMR are presented in Table 1. Cows (n = 16) in treatment sequence A received CON in period 1 and LF in period 2 and averaged 50.8 ± 4.71 kg with a range in milk yield between 40.7 and 59.7 kg/d during the preliminary period. Cows (n = 16) in treatment sequence B received LF in period 1 and CON in period 2 and averaged 50.8 ± 4.16 with a range in milk yield between 42.9 and 58.8 kg/d during the preliminary period.

**Table 1:** Ingredient and nutrient composition of treatment diets

Item	Treatment^1^	
	CON	LF
Ingredient, % DM		
Corn silage	38.8	28.0
Alfalfa silage	14.6	6.16
Alfalfa hay	2.17	2.41
Beet pulp	1.45	9.20
Ground corn	3.74	17.24
High-moisture corn	8.82	—
Corn gluten	1.30	4.86
Soybean meal	7.98	6.57
Soy hulls	5.79	6.93
Cottonseed	5.49	8.13
Vitamin and mineral mix^2^	1.74	1.78
Mineral mix^3^	0.48	0.47
CON protein blend^4^	7.72	—
LF protein blend^5^	—	6.79
C16:0-enriched FA supplement^6^	—	1.43
Nutrient composition, % DM		
DM,^7^ % (as fed)	51.3	61.9
NDF	29.9	30.5
Forage NDF	19.2	12.7
CP	16.3	16.0
RDP	11.0	10.4
RUP	6.2	5.9
MP^8^	11.1	11.0
Lys, % MP	6.45	7.05
Met, % MP	1.78	2.38
Starch	26.6	26.8
FA	2.43	4.21
16:0	0.48	1.76
18:0	0.05	0.15
*cis*-9 18:1	0.42	0.60
*cis*-9,*cis*-12 18:2	1.27	1.54
*cis*-9,*cis*-12,*cis*-15 18:3	0.15	0.09

1 Treatments were (1) control (CON) diet containing high forage and no supplemental fat or supplemental AA; and (2) low-forage (LF) diet containing low forage including supplemental fat and supplemental AA.

2 Vitamin and mineral mix contained 27.1% calcium carbonate, 22.2% calcium phosphate dihydrate, 16.3% ground corn, 15.4% magnesium oxide, 9.6% salt, 4.8% sodium carbonate, 1.7% selenium, and <1% of each of the following: soybean oil, Availa-4 (zinc, manganese, copper, and cobalt mixture, Zinpro, Eden Prairie, MN), manganese sulfate, zinc sulfate, selenium yeast, copper sulfate, cobalt carbonate, 9.2% ethylenediamine dihydroiodide (Vedco Inc., Saint Joseph, MO), vitamin E, vitamin A, and vitamin D_3_ 500 (Baltivet, Dubingai, Lithuania).

3 DCAD Plus (dietary cation-anion difference, Arm & Hammer, Swedesboro, NJ) containing 88.0% DM, 56.0% potassium, and <0.01% of the following: calcium, phosphorus, magnesium, chlorine, sodium, sulfur, cobalt, copper, iodine, iron, manganese, selenium, zinc.

4 CON mix contained 42.3% Amino Plus (Ag Processing Inc., Omaha, NE), 33.1% corn grain, 11.2% sodium sesquinate refined, 6.7% calcium carbonate, 3.9% DCAD Plus (Arm & Hammer, Swedesboro, NJ) 1.5% urea, 1.2% QLF 68 5 Custom (Quality Liquid Feeds, Dodgeville, WI).

5 LF mix contained 44.0% corn grain, 13.2% sodium sequinate refined, 10.6% bypass protein (Caledonia Farmers Elevator), 8.7% calcium carbonate, 6.6% DCAD Plus (Arm & Hammer), 5.5% Amino Plus (Ag Processing Inc., Omaha, NE), 4.9% urea, 3.6% AjiPro L (Ajinomoto Health & Nutrition North America Inc., Chicago, IL), 1.6% molasses (Quality Liquid Feeds, Dodgeville, WI), 1.2% Smartamine M (Adisseo, Alpharetta, GA).

6 Spectrum Fusion (Perdue Agribusiness, Salisbury, MD). Contained (g/100 g of fatty acid; FA) 0.58 of C14:0, 90.2 of C16:0, 0.60 of C18:0, 6.77 of *cis*-9 C18:1, and 93.0% total FA.

7 Expressed as percent of as fed.

8 Calculated using DMI of 30.8 kg/d (CON) and 31.8 kg/d (LF; NRC, 2001).

Dry matter concentrations were determined twice weekly for forages and diets were adjusted accordingly. Diets were mixed separately daily in a mixer wagon. Cows were milked twice daily (0400 and 1500 h) and housed in tiestalls throughout the experiment. Stalls were bedded with sawdust and cleaned twice daily. Access to feed was restricted from 0800 to 1000 h for collection of orts and administration of new feed. Cows were fed at 1000 h daily at 115% expected intake, with water available ad libitum in each stall.

Samples and data for production results were collected during the last 5 d of each treatment period (d 24 to 28). During this time, samples of all diet ingredients (0.5 kg) and orts from each cow (1.0 kg) were collected daily and composited by period for analysis. Milk yield was recorded and samples were collected at each milking as described by Western et al. (2020). Blood (∼15 mL) samples were collected every 15 h resulting in 8 samples/cow per period representing every 3 h over a 24-h period to account for diurnal variation. Blood samples were stored and composited as described by Western et al. (2020). Body weight was measured for each cow 3 times a week for the duration of the trial. On the last day of each period 3 trained investigators determined BCS on a 5-point scale in 0.25 increments. Samples of feed ingredients and orts were processed and analyzed for ash, NDF, CP, starch, and FA concentrations and plasma insulin concentrations as described by Boerman et al. (2015). Individual milk samples were analyzed for fat, true protein, and lactose concentrations and composited based on milk fat yield (d 24–28 of each period) for analysis of FA composition also as described by Boerman et al. (2015).

All data were analyzed using the mixed model procedure of SAS (version 9.4, SAS Institute Inc., Cary, NC) according to the following model:

Y_ijk_ = μ + C_i_ + P_j_ + T_k_ + P_j_T_k_ + e_ijk_,

where Y_ijk_ = the dependent variable, μ = the overall mean, C_i_ = the random effect of cow (i = 1 to 32), P_j_ = the fixed effect of period (j = 1 to 2), T_k_ = the fixed effect of treatment (k = 1 to 2), P_j_T_k_ = the interaction between period and treatment, and e_ijk_ = the residual error. The interaction between period and treatment was removed for all variables when it was not significant (*P* > 0.15). Normality of the results were tested using box plots, normal probability, and homogeneity of variances. Main effects were declared significant at *P* ≤ 0.05 and tendencies at 0.05 < *P* ≤ 0.10. All data were expressed as least squares means and standard error of means, unless otherwise specified.

Treatment diets were similar in NDF, starch, and CP content, and differed in the content of DM, forage NDF, and total FA (Table 1). The CON diet contained 51.3% DM, 19.2% forage NDF, 11.0% RDP, 6.2% RUP, and 2.43% FA, whereas the LF diet contained 61.9% DM, 12.7% forage NDF, 10.4% RDP, 5.9% RUP, and 4.21% FA (Table 1). Compared with CON, LF increased C16:0, C18:0, and *cis*-9 C18:1 by an additional 1.29%, 0.09%, and 0.17% DM, respectively, and increased methionine and lysine by an additional 0.60% and 0.60% MP, respectively.

Compared with CON, the LF diet increased the yields of milk fat (0.06 kg/d; *P* = 0.02), milk protein (0.09 kg/d; *P* < 0.01), 3.5% FCM (1.4 kg/d; *P* = 0.01), and ECM (1.9 kg/d; *P* < 0.01; Table 2). The LF diet also increased DMI (1.00 kg/d; *P* < 0.01), BCS (0.06; *P* = 0.02), and tended to increase BW change (0.20 kg/d; *P* = 0.09) and BCS change (0.06; *P* = 0.08). Compared with CON, the LF diet decreased the content of milk lactose by 0.09 percentage units (*P* < 0.01) and increased milk protein by 0.17 percentage units (*P* < 0.01). No treatment differences were observed for milk yield, lactose yield, fat content, or BW (all *P* > 0.30). Compared with CON, the LF diet increased plasma insulin concentration (*P* < 0.01; Table 2)

**Table 2:** Dry matter intake, milk yield, milk composition, BW, and BCS for cows fed treatment diets (n = 32)

Variable	Treatment^1^		SEM	*P*-value^2^ Trt
	CON	LF		
DMI, kg/d	30.8	31.8	0.40	<0.01
Yield, kg/d				
Milk	45.4	46.1	0.92	0.34
Fat	1.78	1.84	0.04	0.01
Protein	1.47	1.56	0.03	<0.01
Lactose	2.23	2.22	0.05	0.81
3.5% FCM^3^	48.5	49.9	0.89	0.01
ECM^4^	48.3	50.2	0.87	<0.01
Milk composition				
Fat, %	3.95	3.99	0.09	0.37
Protein, %	3.24	3.41	0.04	<0.01
Lactose, %	4.93	4.84	0.02	<0.01
ECM/DMI	1.58	1.55	0.02	0.25
BW, kg	704	703	9.41	0.83
BW change,* kg	0.20	0.40	0.09	0.09
BCS	3.24	3.30	0.06	0.02
BCS change*	0.02	0.08	0.02	0.08
Plasma insulin, μg/L	0.64	0.72	0.03	<0.01

1 Treatments were (1) control (CON) diet containing high forage and no supplemental fat or supplemental AA; and (2) low-forage (LF) diet containing low forage including supplemental fat and supplemental AA.

2 *P*-values associated with treatment (trt).

3 3.5% FCM = [(0.4324 × kg milk) + (16.216 × kg milk fat)].

4 ECM = [(0.324 × kg milk) + (12.95 × kg milk fat) + (7.20 × kg milk protein)]. This equation corrects milk to a 0.68 Mcal/kg energy basis.

* Significant (*P* ≤ 0.10) period × trt interaction.

The yields and contents of milk fat according to source are shown in Table 3. Compared with CON, the LF diet decreased the yield of de novo FA (15 g/d; *P* = 0.03) and increased the yields of mixed FA (44 g/d; *P* < 0.01) and preformed FA (25 g/d; *P* < 0.01). The LF diet increased mixed milk FA mainly due to an increase in the yield of C16:0 in milk fat (*P* < 0.01). The LF diet increased preformed milk FA mainly due to an increase in the yield of unsaturated 18 carbon FA (*P* < 0.01). Contents of milk FA followed the same pattern as yields.

**Table 3:** Fatty acid (FA) contents and yields by source of milk FA for cows fed treatment diets (n = 32)

Variable	Treatment^1^		SEM	*P*-value^2^ Trt
	CON	LF		
Summation by source, g/100 g of FA				
De novo	27.1	25.4	0.27	<0.01
Mixed	39.2	40.6	0.39	<0.01
Preformed	33.6	34.1	0.42	0.03
Summation by source, g/d				
De novo	454	439	11.8	0.03
Mixed	657	701	19.5	<0.01
Preformed	558	583	9.47	<0.01

1 Treatments were (1) control (CON) diet containing high forage and no supplemental fat or supplemental AA; and (2) low-forage (LF) diet containing low forage including supplemental fat and supplemental AA.

2 *P*-values associated with treatment.

With changes in availability of high-quality forages due to unusual cropping seasons, geographic location, or increasing herd size with fixed forage inventories, it is important to explore the effects of lower forage and alternative fiber sources on milk production. Typically, studies investigating the effects of low-forage diets alter the proportion of byproducts, starch, or NDF, and some have supplemented fat to increase the energy density of the ration and support milk fat production. In our current study, to support milk production in low-forage diets we used rumen-protected methionine and lysine and supplemental FA. To our knowledge, no studies have compared the effects of a low-forage diet with FA and AA supplementation with a traditional Midwestern diet fed to high-producing cows.

Variable responses to low-forage diets have been observed, depending on source of nonforage fiber and other dietary factors. Many studies have altered the supply of critical nutrients between low- and high-forage diets, such as total NDF and starch content (Farmer et al., 2014; Hall and Chase, 2014). These studies observed increases in DMI without changes in milk production, resulting in a decrease in feed efficiency in the low-forage treatments. Other studies have tried to increase milk production through the addition of supplemental fat in low-forage diets with variable results (Weiss and Pinos-Rodríguez, 2009; Piantoni et al., 2015).

Our aim was to evaluate if milk component yields could be maintained or increased in a low-forage diet with additional FA and AA supplementation compared with a traditional Midwestern diet. To support milk component production, we recognized the importance of maintaining rumen health by providing enough NDF, starch, and protein in the diet. In our study, dietary forage content was reduced from 55.5% diet DM to 36.6% diet DM, and forage NDF was reduced from 19.2% to 12.7% diet DM. We achieved this by replacing forage (corn silage and alfalfa silage) with nonforage fiber sources (beet pulp, soyhulls, and cottonseed). We replaced high-moisture corn with ground corn to decrease the supply of rapidly fermentable starch in the LF treatment to minimize risk of milk fat depression. Additionally, we altered the amount of ground corn and soybean meal between treatments to balance the supply of starch and CP, and supplemented FA and AA to support the production of milk and milk components in the LF treatment. We used a C16:0-enriched FA supplement due to recent research showing that C16:0 supplementation increases milk yield, milk fat yield, and NDF digestibility compared with other FA supplements and non-FA-supplemented control diets fed to mid-lactation cows (dos Santos Neto et al., 2021b). We maintained RUP while increasing methionine and lysine available for absorption in the LF treatment and decreased RDP to keep CP values similar to the CON treatment. This allowed for the LF treatment to have an increased supply of methionine and lysine to support milk protein synthesis without oversupplying protein and potentially decreasing nitrogen efficiency.

The LF treatment increased DMI, which is similar to previous results with low-forage diets (Clark and Armentano, 1997; Mooney and Allen, 1997). In low-starch diets (21% of DM), replacing dietary forage with byproducts increased DMI (Farmer et al., 2014). Similarly, Weiss and Pinos-Rodríguez (2009) observed that cows fed wheat middlings and soybean hulls in partial replacement of corn silage and alfalfa increased DMI compared with a high-forage diet. Increased DMI in low-forage diets can be attributed to a decreased supply of forage NDF and smaller particle length, leading to a decrease in physical fill and rumen retention rate (Allen, 2000). In addition, nonforage fiber sources increase DMI by increasing NDF digestibility (Mooney and Allen, 1997). While we did not measure rumination or average particle length, the effects of low-forage diets on these variables have been well studied in previous research (Allen, 2000). Although fat supplementation has variable effects on DMI depending on FA profile (Allen, 2000; dos Santos Neto et al., 2021a,b), a recent meta-analysis observed that when C16:0-enriched supplements were fed at <3% of diet DM they had no effect on DMI compared with non-FA-supplemented control diets (dos Santos Neto et al., 2021b). Therefore, C16:0 supplementation in LF in the current study likely did not influence or contribute to the observed increase in DMI.

Although we observed no effect of treatments on milk yield, LF increased ECM yield because it increased the yields of both milk fat and protein compared with CON. Under typical forage conditions, C16:0 supplementation increases milk fat yield compared with non-FA-supplemented control diets (dos Santos Neto et al., 2021b). C16:0 supplementation in LF likely supported the increase in milk fat yield by providing additional C16:0 for milk fat synthesis. Although most of our studies involving C16:0-enriched supplements (fed at <2.0% diet DM) have observed increases in ECM yield (e.g., de Souza et al., 2018), these increases were driven by milk fat responses while milk protein yield was unaffected. However, increases in milk protein yield were observed with C16:0 supplementation compared with a non-FA-supplemented control diet and other FA supplements in studies where the basal diet contained high-quality blood meal (de Souza et al., 2019; Western et al., 2020). The LF diet increased milk protein yield, likely partially due to increases in DMI providing adequate energy from starch for microbial protein production, combined with additional methionine and lysine supplying more nutrients for milk protein synthesis in the mammary gland. The increase in insulin concentration observed with LF could also support milk protein synthesis in the mammary gland (Mackle et al., 1999; Winkelman and Overton, 2013). Mackle et al. (1999) used a hyperinsulinemic-euglycemic clamp with or without infusions of casein plus branched-chain AA and observed that insulin by itself increased milk protein yields by 15%, and when combined with abomasal infusion of casein plus branched-chain AA, milk protein yield increased by 25%. However, infusion of casein plus branched-chain AA without the insulin clamp did not affect the concentration or yield of milk protein (Mackle et al., 1999).

Fatty acid supplementation alters milk FA content and yield and is affected by the FA being supplemented. In a recent meta-analysis, dos Santos Neto et al. (2021b) observed that supplementation of C16:0 increased total milk FA, primarily due to an increase in mixed-source FA. The reduction of de novo milk FA yields in our trial agrees with responses to a C16:0-enriched FA supplement observed in some trials (Western et al., 2020), but not others (de Souza and Lock, 2018). The increase in DMI with LF, coupled with the higher dietary FA content in the diet, provided more long-chain FA for incorporation into milk FA. Similarly, rumen-protected methionine and lysine supplementation to Comisana ewes increased 16-carbon FA concentrations in milk fat (Sevi et al., 1998). Overall, the milk FA responses we observed with LF were as expected when increasing C16:0 and total dietary FA content.

A factorial design would be needed to test the specific effects of FA and AA supplementation, but this was not the goal of our study. Rather, our focus was to determine if we can formulate low-forage diets to increase yields of milk fat and protein. We demonstrated that low-forage diets can be formulated to increase ECM compared with traditional Midwestern diets. Accounting for the change in DMI between the 2 treatments, cows on LF consumed 5.5 kg/d less forage DM than cows on CON. Over 28 d, this equated to ∼150 kg less forage DM fed per cow. However, long-term studies are required to determine if feeding low-forage diets can maintain healthy BW, rumen health, and productivity for long-term implementation in the industry. Further research could also examine the importance of AA and FA supplementation when using low-forage diet strategies.

In high-producing dairy cows, a diet containing only 13% forage NDF supplemented with a C16:0-enriched FA supplement and rumen-protected methionine and lysine increased DMI and the yields of milk fat and protein compared with a control diet containing 19% forage NDF. Cows on the LF treatment consumed 5.5 kg/d less forage DM compared with CON yet maintained milk yield and increased ECM yield. Under certain circumstances where forage inventories are limited due to increasing cow numbers or unusual cropping seasons, low-forage diets can be formulated to sustain, or even increase, milk component yields in high-producing cows.
